# Frame-Rate-Independent High-Frequency 3D-DIC Vibration Measurement Enabled by Stroboscopic Equivalent-Time Sampling and Physics-Guided Spatiotemporal Filtering

**DOI:** 10.3390/s26196164

**Published:** 2026-09-29

**Authors:** Chao Li, Penglong Wang, Zhipeng Sheng, Shizhan Chen, Zhan Huang, Jixu Zhang, Zeren Gao, Yu Fu

**Affiliations:** 1 Lenovo (Shanghai) Information Technology Co., Ltd., No. 696 Songtao Road, Shanghai 201203, China; 2 Shenzhen Key Laboratory of Intelligent Optical Measurement and Detection, College of Physics and Opto-Electronic Engineering, Shenzhen University, 3688 Nanhai Avenue, Shenzhen 518060, China; chenshizhan07@163.com (S.C.); gaozeren@szu.edu.cn (Z.G.); fuyuoptics@gmail.com (Y.F.); 3 State Key Laboratory of Radio Frequency Heterogeneous Integration, Shenzhen University, 3688 Nanhai Avenue, Shenzhen 518060, China; 4 Tech Academy, Shenzhen Polytechnic University, Shenzhen 518060, China

**Keywords:** three-dimensional digital image correlation, full-field vibration measurement, stroboscopic equivalent-time sampling, spatiotemporal noise decoupling, high-frequency sensing, modal analysis

## Abstract

High-frequency full-field vibration measurement using three-dimensional digital image correlation (3D-DIC) is limited by the trade-off between camera frame rate, spatial resolution, and measurement noise. This study presents a method to overcome the frame-rate limitation of 3D-DIC vibration measurement by combining stroboscopic equivalent-time sampling with spatiotemporal noise decoupling. Short-pulse stroboscopic illumination freezes structural motion at different vibration phases, allowing a low-frame-rate stereo camera system to reconstruct high-frequency periodic responses through inter-cycle phase sampling. The measured three-dimensional displacement fields are further processed as space–time data, where target-frequency extraction and spatial-frequency filtering are combined to suppress noise and enhance small-amplitude vibration responses. The proposed method was experimentally validated using a plastic plate vibrating at 251.1 Hz. With an actual camera frame rate of approximately 8.34 fps and an equivalent temporal sampling frequency of 2511 Hz, the reconstructed mode shape achieved a Modal Assurance Criterion (MAC) value of 0.9233, comparable to that obtained using a high-speed camera (0.9225), while providing higher spatial resolution and lower hardware cost. A pulse-width experiment on an aluminum plate vibrating at 7154 Hz demonstrated the influence of stroboscopic exposure duration on measurement accuracy. The proposed approach was further integrated into the EMODE-1 full-field vibration measurement system and applied to a Lenovo ThinkPad touchpad vibrating at 1051 Hz, achieving a MAC value of 0.8878 compared with continuous-scanning laser Doppler vibrometry. The results demonstrate the potential of the proposed method for high-frequency full-field vibration sensing of periodic structures using compact and cost-effective imaging systems.

## 1. Introduction

Structural vibration plays a critical role in the design, reliability assessment, and health monitoring of engineering systems. Under cyclic loading, resonance, or random excitation, excessive vibration may induce fatigue damage, crack propagation, connection failure, and even catastrophic structural failure [1,2]. Experimental modal analysis is therefore widely used to identify natural frequencies, damping characteristics, and mode shapes for validating numerical models and evaluating dynamic performance [3,4]. Since mode shapes contain both temporal-frequency and spatial-distribution information, advanced vibration measurement techniques are required to simultaneously provide high temporal resolution, dense spatial sampling, and high measurement accuracy, particularly for lightweight, complex, and high-frequency structures [5,6,7,8].

Optical measurement techniques have been extensively investigated for vibration characterization due to their noncontact nature and high sensitivity. Interferometric methods, including holographic interferometry [9], electronic speckle pattern interferometry [10], and shearography [11], can achieve submicrometer or even nanometer-scale sensitivity, but they generally require high optical stability and often provide limited time-resolved information. Laser Doppler vibrometry (LDV) provides excellent temporal resolution, broadband capability, and noncontact measurement by detecting Doppler frequency shifts of scattered light [12]. However, conventional LDV systems are primarily point-based measurements, and scanning laser Doppler vibrometry (SLDV) [13] or continuous-scanning laser Doppler vibrometry (CSLDV) [14] methods, although capable of obtaining vibration mode shapes, are limited by scanning time, optical accessibility, and measurement efficiency for complex structures [15,16].

Digital image correlation (DIC) has emerged as an attractive alternative for vibration measurement because it provides full-field displacement measurement with dense spatial sampling and flexible experimental configurations [17]. By employing high-resolution cameras and stereo vision, three-dimensional DIC (3D-DIC) can reconstruct surface deformation and vibration mode shapes without requiring scanning mechanisms. However, high-frequency vibration measurement using 3D-DIC faces a fundamental limitation caused by the trade-off between camera frame rate, spatial resolution, and measurement noise. Increasing the frame rate of high-speed cameras usually requires sacrificing image resolution, field of view, and signal-to-noise ratio, while also significantly increasing system cost and data-processing requirements [18]. Therefore, directly increasing the camera frame rate is not always an effective solution for high-frequency full-field vibration sensing.

Stroboscopic equivalent-time sampling provides a potential approach to overcome this limitation by exploiting the periodic repeatability of steady-state vibration signals. Instead of increasing the physical frame rate of the camera, equivalent-time sampling reconstructs high-frequency vibration responses by acquiring different phase states over multiple vibration cycles and rearranging them according to vibration phase [19]. This strategy enables conventional cameras with high spatial resolution to achieve high equivalent temporal resolution. Recent studies have advanced full-field dynamic measurement through low-frame-rate reconstruction and short-exposure imaging. Kato et al. combined sub-Nyquist-rate 3D-DIC with compressed sensing and order analysis for high-frequency micro-vibration measurement under operational conditions [20]. Zhao et al. combined nanosecond-scale time-gated imaging with single-camera split-screen stereo-DIC to suppress motion blur and avoid dual-camera synchronization issues [21]. These developments highlight the distinct but complementary roles of effective exposure control and temporal reconstruction. Despite these advances, small-amplitude vibration measurements using DIC remain susceptible to image noise, speckle quality, correlation errors, and environmental disturbances. Consequently, an effective signal-processing strategy is required to extract weak vibration responses while preserving the spatial characteristics of structural mode shapes.

In this study, a method for overcoming the frame-rate limitation of 3D-DIC vibration measurement is proposed by integrating stroboscopic equivalent-time sampling with spatiotemporal noise decoupling. A stroboscopic 3D-DIC vibration measurement system is developed, where short-pulse illumination is used to freeze structural motion and achieve phase-synchronized stereo image acquisition. The capability of a low-frame-rate stereo camera system to reconstruct high-frequency vibration responses is demonstrated. A spatiotemporal noise-decoupling method is proposed by treating the measured displacement field as three-dimensional space-time data. Temporal-frequency selection is combined with spatial-frequency filtering to suppress non-target components and improve the signal-to-noise ratio of small-amplitude vibration measurements. The proposed approach is systematically validated through comparisons with high-speed camera measurements, investigations of stroboscopic pulse-width effects, and practical applications using the EMODE-1 full-field vibration measurement system on a Lenovo ThinkPad touchpad. The proposed method provides a compact and cost-effective solution for high-frequency full-field vibration sensing, enabling conventional stereo imaging systems to achieve high equivalent temporal resolution without sacrificing spatial resolution.

## 2. Principle

### 2.1. Principle of 3D-DIC Vibration Measurement

**Figure 1:**
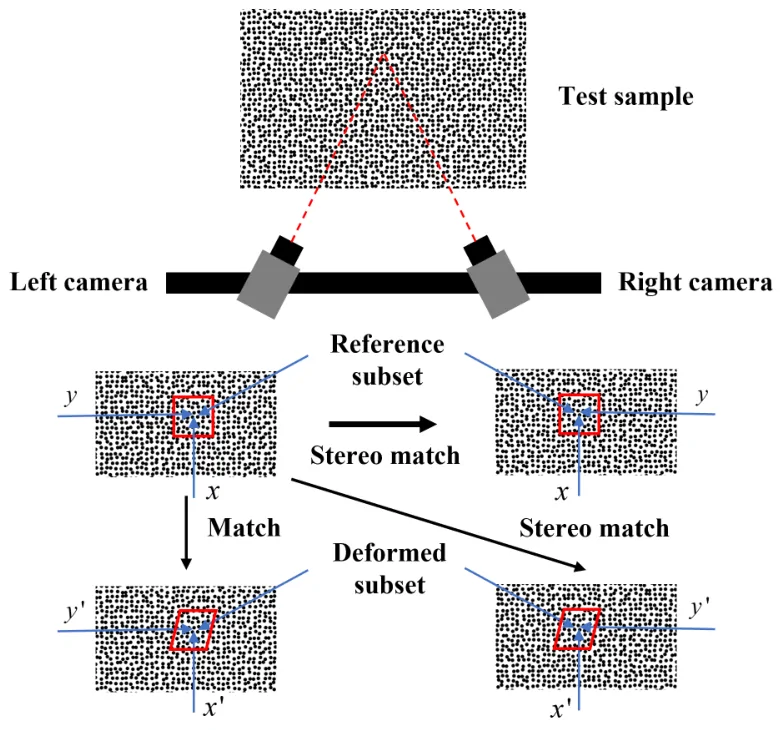
Schematic of the 3D-DIC principle.

3D-DIC uses two cameras to synchronously image the test surface from different viewpoints and reconstruct its three-dimensional geometry and displacement based on stereo vision, as illustrated in Figure 1. First, the intrinsic and extrinsic parameters of the two cameras are obtained through stereo calibration [22]. The undeformed image is then defined as the reference image, and the deformed image as the target image. A reference subset f(x,y), centered at the point of interest, is established in the reference image, and the most similar deformed subset $g(x^{\prime },y^{\prime })$ is searched for in the target image. The zero-mean normalized sum of squared differences (ZNSSD) criterion is used to evaluate the similarity between the two subsets:

(1) $$C_{\mathrm{ZNSSD}}={\left[\frac{\sum_{y=-M}^{M}\sum_{x=-M}^{M}f\left(x,y\right)-\bar{f}}{\sqrt{\sum_{y=-M}^{M}\sum_{x=-M}^{M}{\left[f\left(x,y\right)-\bar{f}\right]}^{2}}}-\frac{\sum_{y^{\prime }=-M}^{M}\sum_{x^{\prime }=-M}^{M}g\left(x^{\prime },y^{\prime }\right)-\bar{g}}{\sqrt{\sum_{y^{\prime }=-M}^{M}\sum_{x^{\prime }=-M}^{M}{\left[g\left(x^{\prime },y^{\prime }\right)-\bar{g}\right]}^{2}}}\right]}^{2},$$

where *M* denotes the half-width of the subset; $\bar{f}$ and $\bar{g}$ denote the mean grayscale values of the reference and deformed subsets, respectively. The inverse compositional Gauss–Newton (ICGN) algorithm is used to iteratively solve for the optimal parameters of the correlation function. Because a subset may undergo translation, stretching, and shear simultaneously, a first-order shape function is adopted to describe the mapping between the reference and deformed subsets:

(2) $$\left[\begin{array}{c} x^{\prime }\\ y^{\prime } \end{array}\right]=\left[\begin{array}{ccc} 1+u_{x} & u_{y} & u\\ v_{x} & 1+v_{y} & v \end{array}\right]\left[\begin{array}{c} \Delta x\\ \Delta y\\ 1 \end{array}\right],$$

where Δx and Δy are the coordinate increments of the calculation point relative to the subset center (x,y); *u* and *v* are the horizontal and vertical displacements of the subset center, respectively, and $u_{x}$, $u_{y}$, $v_{x}$, and $v_{y}$ are the corresponding displacement gradients. Once the iteration satisfies the convergence criterion, the subpixel displacements in the left and right camera images are obtained. Using the stereo calibration parameters and the stereo matching relationship, the three-dimensional coordinates and displacement field of the measured surface can then be calculated.

### 2.2. Principle of 3D-DIC Stroboscopic Equivalent-Time Sampling

**Figure 2:**
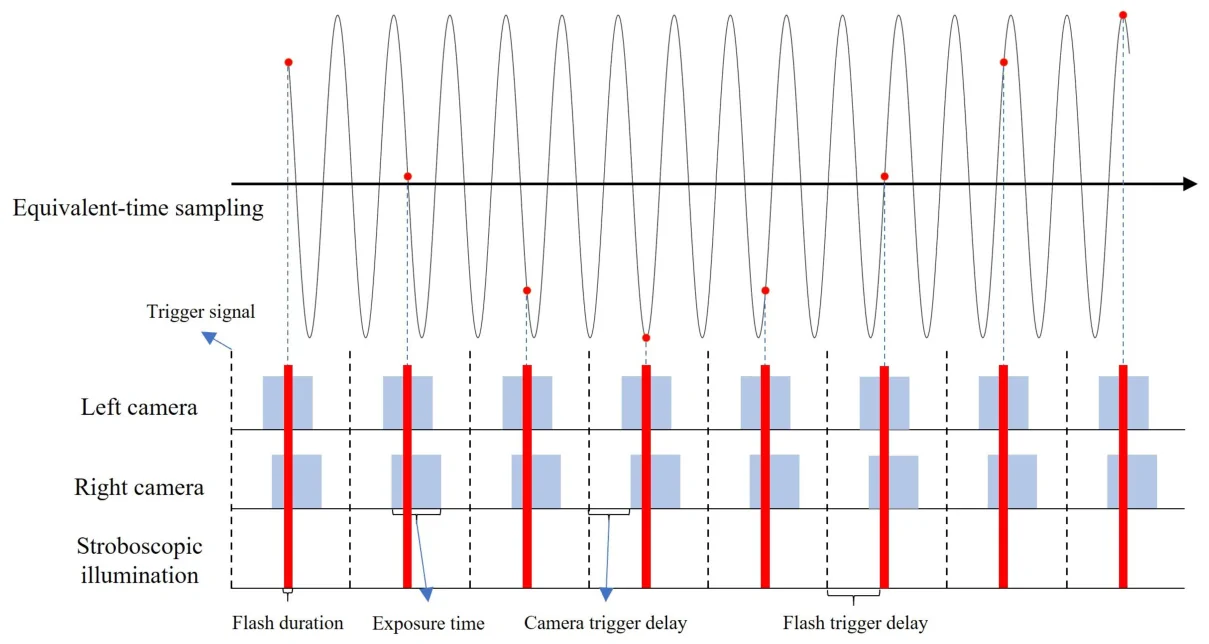
Schematic of stroboscopic equivalent-time sampling.

Equivalent-time sampling is applicable to periodic vibrations with stable frequencies and repeatable responses. Its principle is illustrated in Figure 2. For a single-frequency signal with frequency *f* and period T=1/f, if *n* equally spaced phase samples are specified within one period, the time interval between adjacent equivalent samples is Δt=T/n. The camera acquires the next phase sample after *k* complete vibration periods plus one phase increment Δt, thereby implementing inter-cycle signal sampling. Therefore, the interval between two consecutive actual exposures is

(3) $$T_{s}=kT+\Delta t=kT+\frac{T}{n},$$

The corresponding actual camera sampling rate is

(4) $$f_{s}=\frac{1}{T_{s}}=\frac{fn}{kn+1},$$

where *k* and *n* are positive integers. With one effective illumination pulse per acquired image, sampling at the vibration frequency or an integer submultiple of it repeatedly captures the same phase and cannot, by itself, reconstruct the complete periodic response. Sampling at an integer multiple *m* of the vibration frequency provides *m* discrete phases per cycle if each pulse is recorded separately. In contrast, the interval $T_{s}$ used here introduces a phase increment of 2π/n between successive samples, allowing *n* uniformly spaced phases to be acquired over repeated vibration cycles. After acquisition, the images are rearranged according to the vibration phase for each frame, yielding an equivalent sequence with an equivalent sampling rate of n×f. This method does not circumvent the Nyquist–Shannon sampling theorem [23]; rather, it exploits the repeatability of a steady periodic response to assemble samples acquired over multiple cycles into an equivalent-time sequence. For the single-frequency steady-state response considered here, the equivalent sampling rate n×f must likewise exceed twice the vibration frequency *f*. Therefore, the vibration response must remain sufficiently repeatable, and the prescribed sampling-phase relationship must be maintained throughout acquisition. Meanwhile, if the camera exposure time is excessively long relative to the vibration period, intra-period motion still causes image blur, and inconsistent trigger delays between the stereo cameras may introduce phase errors. Short-pulse stroboscopic illumination is therefore used to define the effective exposure and synchronize the stereo cameras.

Stroboscopic acquisition synchronizes short-pulse illumination with a specified phase of the vibrating object under unified timing control, so that the cameras capture vibration information only within a very short illumination window, forming the effective images. As illustrated in Figure 2, during the camera exposure time $T_{\mathrm{cam}}$, the stroboscopic light source emits a high-intensity pulse of width $T_{\mathrm{flash}}$, much shorter than the vibration period. In this case, $T_{\mathrm{flash}}$ can be considered the system’s effective exposure time. Provided that the image-plane displacement during the pulse is smaller than the blur tolerance of DIC, the instantaneous state of the specimen can be approximately frozen. For a stereo system, even when the two cameras differ in electronic trigger delay and exposure start time, the two images correspond to the same vibration phase as long as the stroboscopic pulse lies within the overlap of the two exposure windows, thereby achieving optical-level synchronized acquisition.

### 2.3. Spatiotemporal Noise-Decoupling Method for 3D-DIC

**Figure 3:**
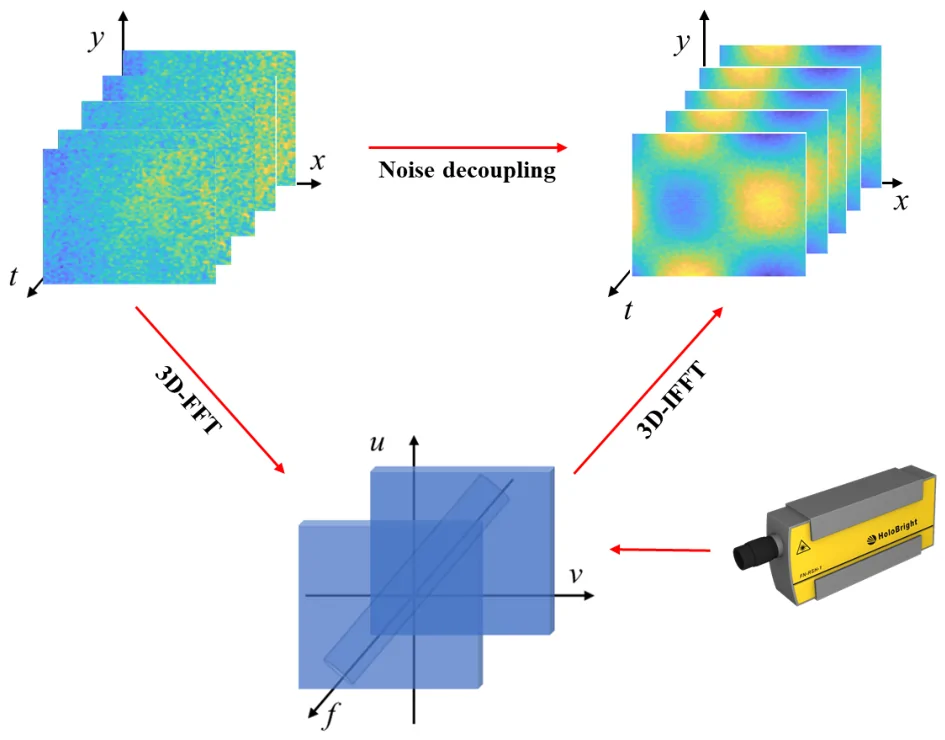
Schematic of the three-dimensional noise-decoupling method. In the illustrative displacement maps, yellow denotes positive displacement and blue denotes negative displacement. The maps are normalized only to show the mode-shape pattern, not absolute displacement.

The continuous 3D-DIC measurement results can be represented as three-dimensional spatiotemporal data: each frame describes the full-field displacement at a given instant, while consecutive frames constitute the vibration time histories at all spatial points. For small-amplitude vibrations, the target response is readily affected by random image noise, correlation errors, and environmental disturbances. To exploit both the temporal-frequency and spatial-distribution characteristics of vibration, the three-dimensional noise-decoupling method shown in Figure 3 is proposed. First, a three-dimensional Fourier transform is applied to the DIC displacement data f(x,y,t), yielding the frequency-domain representation F(u,v,f):

(5) $$F\left(u,v,f\right)=\int_{-\infty }^{\infty }\int_{-\infty }^{\infty }\int_{-\infty }^{\infty }f\left(x,y,t\right)e^{-j2\pi (ux+vy+ft)}\;dx\;dy\;dt,$$

where (x,y,t) denotes the spatial and temporal coordinates, and (u,v,f) denotes the corresponding spatial and temporal frequencies. The target vibration frequency $f_{v}$ is determined from the LDV measurement or known excitation information. The target component is then retained along the temporal-frequency axis, while DC drift and other non-target-frequency components are suppressed. The temporal filtering function is

(6) $$H_{t}\left(f\right)=\left\{\begin{array}{cc} 1, & f=f_{v},\\ 0, & \mathrm{else}, \end{array}\right.$$

After temporal-frequency selection, the target-frequency component may still contain random errors that vary rapidly in space. Because structural mode shapes are generally spatially continuous and relatively smooth, their spatial-frequency content is concentrated at low frequencies. A Gaussian low-pass window is therefore further applied in the spatial-frequency dimensions to suppress high-spatial-frequency noise. The corresponding filtering function is

(7) $$H_{s}\left(u,v\right)=G\left(u,v\right),$$

where G(u,v) is the Gaussian low-pass filter kernel in the frequency domain. Temporal-frequency selection and spatial low-pass filtering together constitute the three-dimensional noise-decoupling process. Finally, the target vibration displacement field is reconstructed through the three-dimensional inverse Fourier transform:

(8) $$f^{\prime }\left(x,y,t\right)=\int_{-\infty }^{\infty }\int_{-\infty }^{\infty }\int_{-\infty }^{\infty }F\left(u,v,f\right)\;H_{t}\left(f\right)\;H_{s}\left(u,v\right)e^{j2\pi (ux+vy+ft)}\;du\;dv\;df,$$

## 3. Experimental Results for Method Validation

### 3.1. Comparison Between High-Speed and Low-Speed Cameras

**Figure 4:**
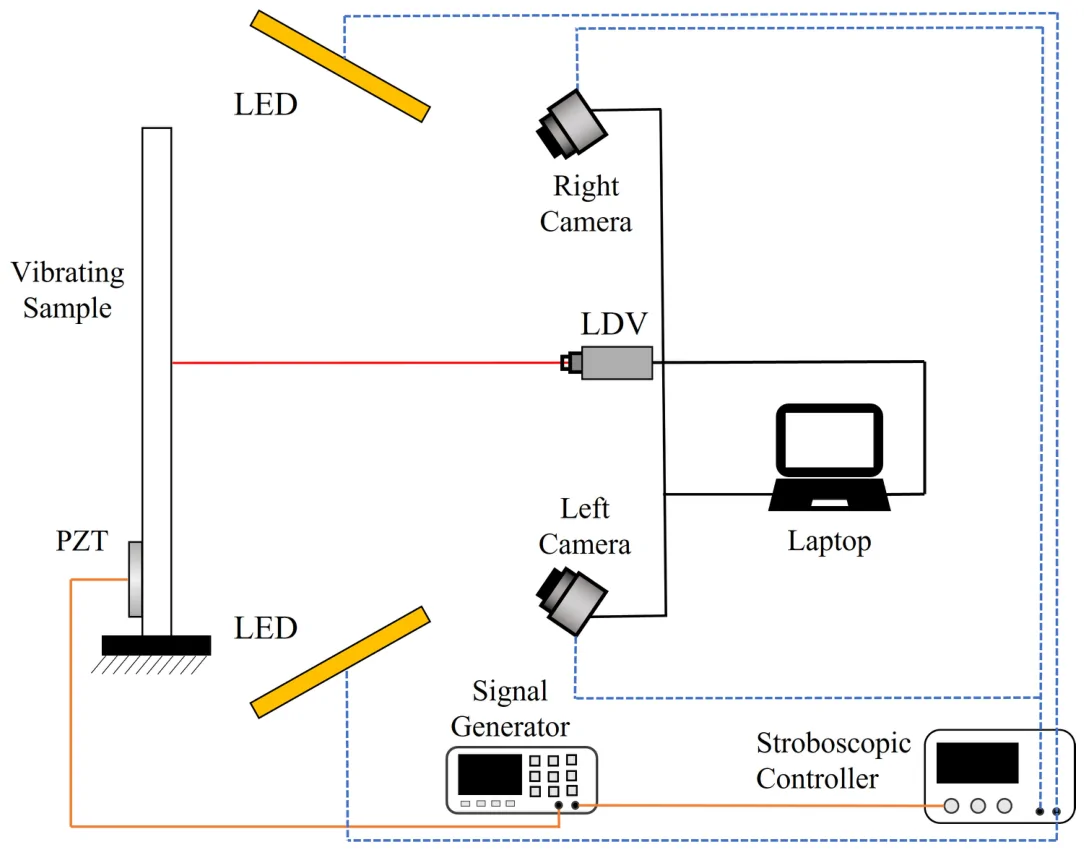
Schematic of the experimental setup for 3D-DIC vibration measurement.

**Figure 5:**
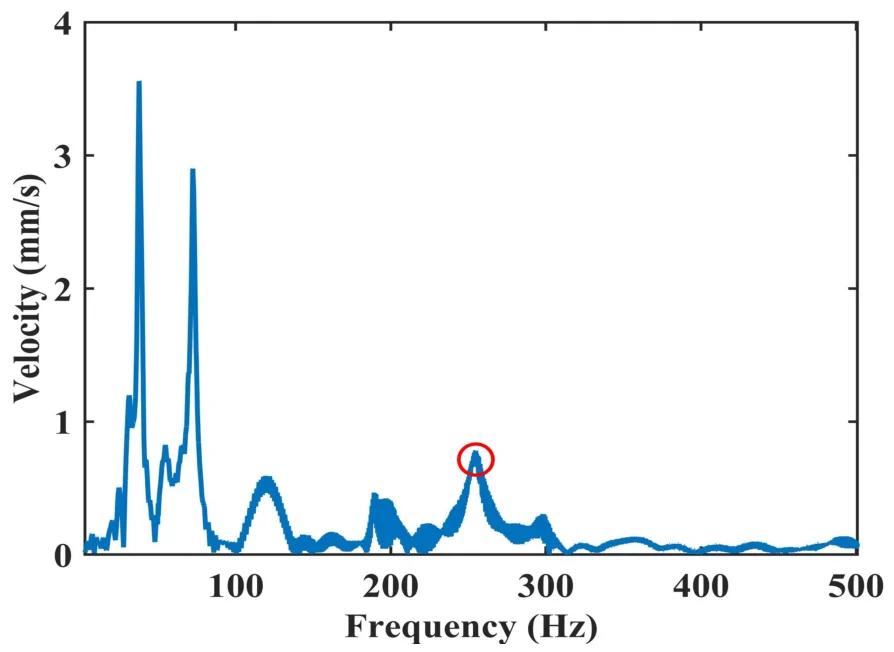
Frequency spectrum of the natural frequencies of the plastic plate. The red circle marks the 251.1 Hz peak selected for single-frequency excitation.

The experimental setup is shown in Figure 4. The test specimen was a 100 mm × 100 mm plastic plate clamped along one edge. A PZT ceramic patch bonded to the rear surface of the plate served as the excitation source, and the excitation signal was generated by a signal generator (SDG6052X, SIGLENT Technologies Co., Ltd., Shenzhen, China). The structural frequency response was first measured using an LDV (PNV-RD-AVD1, HoloBright (S) Pte. Ltd., Singapore), as shown in Figure 5. Based on the response peak, 251.1 Hz was selected as the single-frequency excitation for the subsequent experiments.

Continuous sampling with a high-speed camera (CP70-1HS-M/C-1900, Optronis GmbH, Kehl, Germany) and stroboscopic equivalent-time sampling with a low-speed camera (MV-CH050-10UM, Hikrobot, Hangzhou, China) were performed. The excitation condition, measurement field of view (approximately 160 mm × 160 mm), equivalent phase interval, and number of images were kept identical in the two experiments: 10 phase points were specified per vibration period, and 80 periods were reconstructed, corresponding to 800 stereo image pairs. The high-speed camera acquired continuously at 2511 fps with the LEDs continuously illuminated. For the low-speed camera, whose nominal acquisition rate was approximately 8.342 fps, the signal generator and stroboscopic controller (SP-24W90-2T, Dongguan Chuangshi Automation Technology Co., Ltd., Dongguan, China) synchronously controlled the cameras and light sources according to the equivalent-time sampling sequence. The stroboscopic pulse width was set to 10 μs, and the equivalent sampling rate after temporal reordering was also 2511 Hz. The LDV synchronously measured one point on the plate, serving both as the single-point reference and as the source of the target frequency required for three-dimensional noise decoupling. The main parameters of the two cameras are listed in Table 1. For the low-speed stroboscopic acquisition, the illumination source and cameras were externally triggered by an FPGA operating with a nominal 50 MHz clock, corresponding to a timing resolution of 20 ns. The value of 8.342193 fps in Table 1 is the rounded nominal acquisition rate calculated from the equivalent-time sampling relation, rather than a measured value accurate to six decimal places. The actual trigger-frequency accuracy depends on the timebase accuracy and timing implementation.

**Figure 6:**
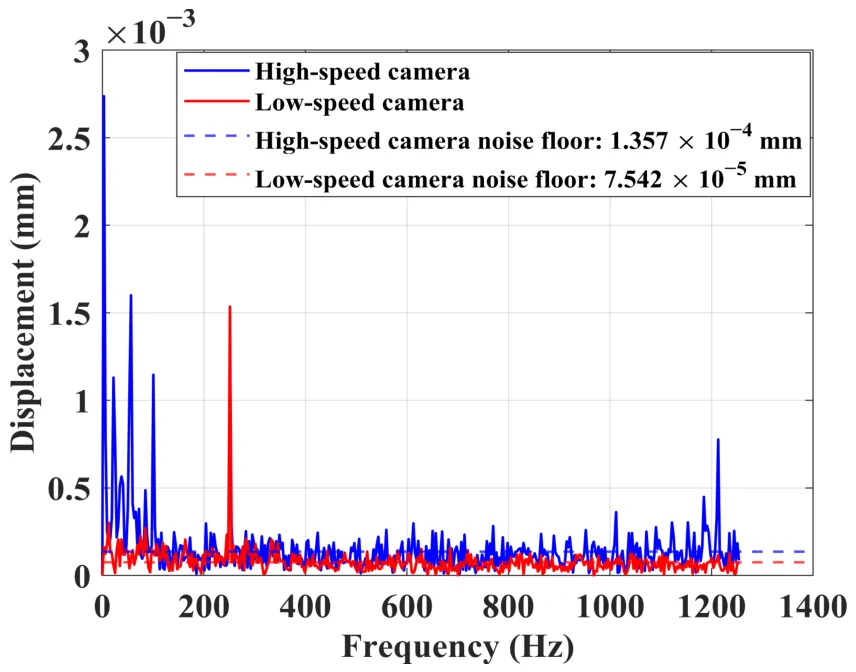
Comparison of the frequency-domain raw single-point DIC measurements obtained using the high-speed and low-speed cameras.

Figure 6 compares the single-sided amplitude spectra of the raw DIC displacements at the LDV measurement point. Both sampling approaches exhibit a distinct peak at 251.1 Hz, indicating that stroboscopic equivalent-time sampling correctly recovers the target frequency. The high-speed camera measurement contained low-frequency noise from 0 to 20 Hz, as well as noise components at 50 and 100 Hz introduced by the LED light source. Therefore, the mean noise levels of the two datasets were calculated after excluding these noise components and the vibration component. The results show that, under the present experimental settings, the low-speed camera had a lower noise level. This result indicates that stroboscopic equivalent-time sampling can preserve the target vibration component while exploiting a conventional high-resolution camera to obtain a favorable signal-to-noise ratio, and therefore has considerable potential for vibration measurement.

**Figure 7:**
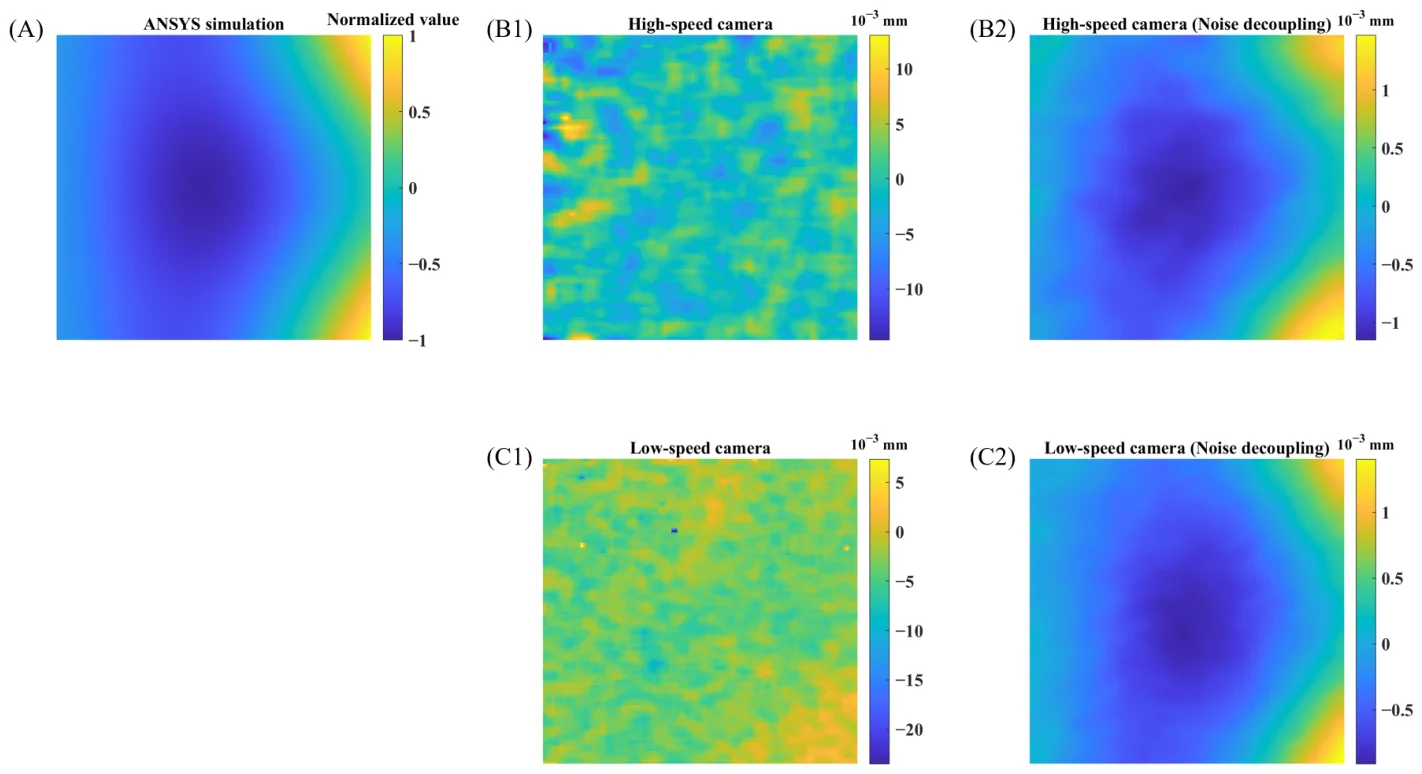
Comparison of mode-shape results. (**A**) ANSYS simulation; (**B1**,**B2**) raw and three-dimensionally noise-decoupled results obtained using the high-speed camera, respectively; (**C1**,**C2**) raw and three-dimensionally noise-decoupled results obtained using the low-speed camera, respectively. (**A**) is normalized solely for comparison of mode-shape patterns; the experimental color bars show displacement in thousandths of a millimeter.

**Figure 8:**
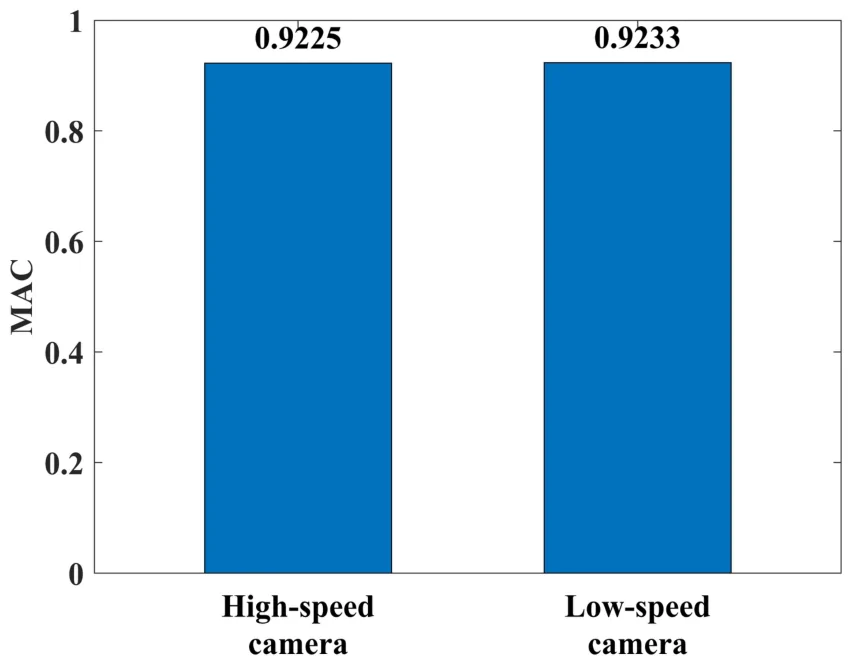
Comparison of the MAC values between the noise-decoupled mode shapes and the simulated mode shape.

Subsequently, the three-dimensional noise-decoupling method was applied to both DIC datasets. Figure 7 presents the mode shape simulated using ANSYS 2025 R1, the raw DIC mode shapes obtained with the two cameras, and the corresponding mode shapes after noise decoupling. In the raw results, spatial random noise obscures the target mode-shape features. After target-frequency selection and spatial low-pass constraint, both measurements recover smooth mode shapes consistent with the simulation. Figure 8 further presents the MAC values, which are 0.9225 and 0.9233 for the high-speed and low-speed cameras, respectively, demonstrating strong agreement between the measured and simulated mode shapes and validating the effectiveness of the three-dimensional noise-decoupling method. The small difference between the two MAC values also indicates that, provided that the vibration is periodic and stable and that the equivalent-time sampling parameters are correctly configured, stroboscopic equivalent-time sampling can achieve mode-shape reconstruction accuracy comparable to that of continuous high-speed sampling.

**Figure 9:**
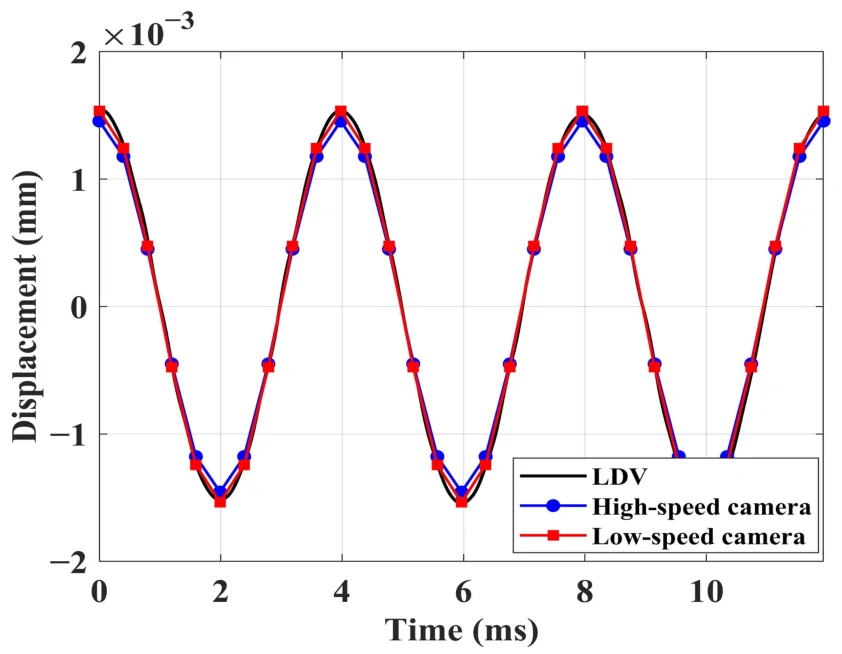
Comparison of the time-domain single-point measurements obtained using the high-speed and low-speed cameras after DIC noise decoupling.

Figure 9 compares the two noise-decoupled DIC displacement results at the LDV measurement point with the LDV reference displacement. Both DIC results agree closely with the LDV in amplitude and phase and successfully recover a displacement response of approximately 1.5 μm, indicating that three-dimensional noise decoupling suppresses noise while preserving the true vibration information at the target frequency. As shown in Table 1, at the same equivalent sampling rate, the low-speed camera provided a higher spatial resolution (1168 × 1200 versus 832 × 840 for the high-speed camera), lower equipment cost, and, in the present experiment, a lower spectral noise floor. Therefore, for periodic vibrations with stable frequencies, stroboscopic equivalent-time sampling can achieve measurement results comparable to those of high-speed sampling at a lower hardware cost, without sacrificing spatial resolution to increase frame rate, thereby broadening the range of vibration-measurement applications.

A fair comparison between the high-speed and low-speed cameras also requires control of the field of view and speckle-image quality. In this experiment, the fields of view of the two cameras were kept at the same scale. Because the low-speed camera had approximately twice as many pixels as the high-speed camera, the same physical speckles occupied approximately 3 pixels in the high-speed images and 6 pixels in the low-speed images, so the two image sets represented similar physical speckle scales. In addition, stroboscopic equivalent-time sampling relies on periodic stability and a known vibration frequency. Frequency drift or trigger-phase errors accumulate during prolonged acquisition. Therefore, the method is applicable to steady-state or highly repeatable periodic vibrations and cannot replace direct high-speed-camera recording of transient, random, or rapidly time-varying processes.

### 3.2. Stroboscopic Pulse-Width Experiment

**Figure 10:**
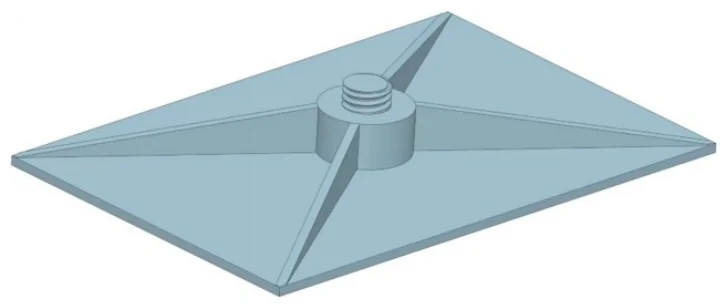
Aluminum plate specimen used in the experiment.

**Figure 11:**
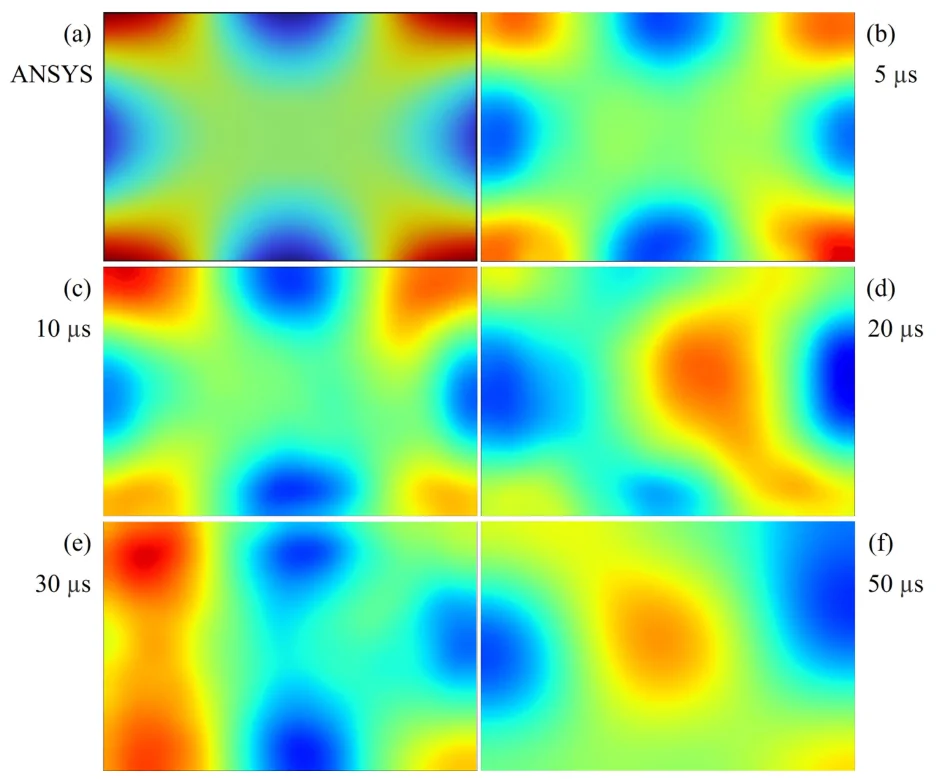
Mode shapes of the aluminum plate obtained with different stroboscopic pulse widths: (**a**) ANSYS result; (**b**–**f**) instrument measurements obtained at pulse widths of 5, 10, 20, 30, and 50 μs, respectively. Red indicates positive displacement and blue indicates negative displacement. The maps are normalized only to show the mode-shape patterns, not absolute amplitudes.

Having verified the measurement accuracy of stroboscopic equivalent-time sampling and three-dimensional noise decoupling, the influence of the stroboscopic pulse width on high-frequency vibration imaging was further investigated. The stroboscopic pulse width determines the effective exposure time of the system and is therefore a key parameter affecting imaging quality. In this experiment, the low-speed stereo-camera system and the stroboscopic light source were used to measure the mode shape of an aluminum plate under 7154 Hz single-frequency excitation, with pulse widths of 5, 10, 20, 30, and 50 μs. The aluminum plate specimen is shown in Figure 10, and the measurement results are presented in Figure 11. At a pulse width of 5 μs, the measured mode shape was closest to the ANSYS result. As the pulse width increased, motion blur became progressively more pronounced, resulting in greater mode-shape distortion. A vibration frequency of 7154 Hz corresponds to a period of approximately 139.8 μs, and a pulse width of 5 μs accounts for approximately 3.6% of one vibration period. These results indicate that the stroboscopic pulse width should be substantially shorter than the vibration period and that the image-plane motion during the light pulse should remain below the blur tolerance of DIC.

The above experiments validate both the measurement accuracy of the proposed method and the key imaging condition required for its implementation. Based on these results, the proposed method was further integrated into a compact measurement system and evaluated in a practical application scenario.

## 4. System Integration and Experimental Validation in a Practical Scenario

**Figure 12:**
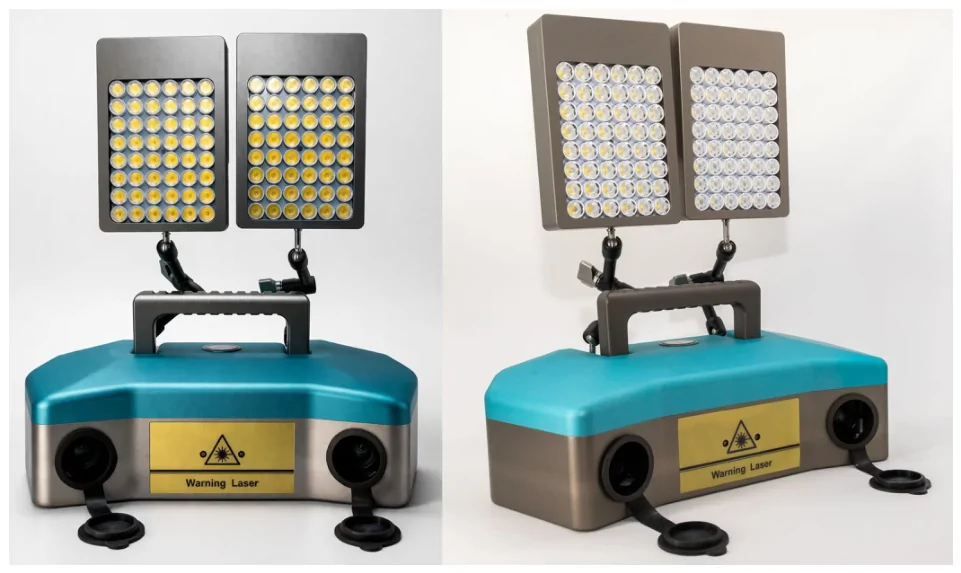
Appearance of the EMODE-1 measurement system.

The experiments presented in Section 3 verified the measurement accuracy of the proposed method and clarified the stroboscopic pulse-width requirement for suppressing motion blur. On this basis, the method was implemented in an integrated measurement system to improve the compactness and operational convenience of the experimental setup. The stroboscopic controller, stroboscopic light sources, and stereo cameras were integrated into a single device, while the stroboscopic equivalent-time sampling and three-dimensional noise-decoupling algorithms were incorporated into the supporting software. The resulting measurement system was named EMODE-1, as shown in Figure 12, and its main specifications are listed in Table 2.

**Figure 13:**
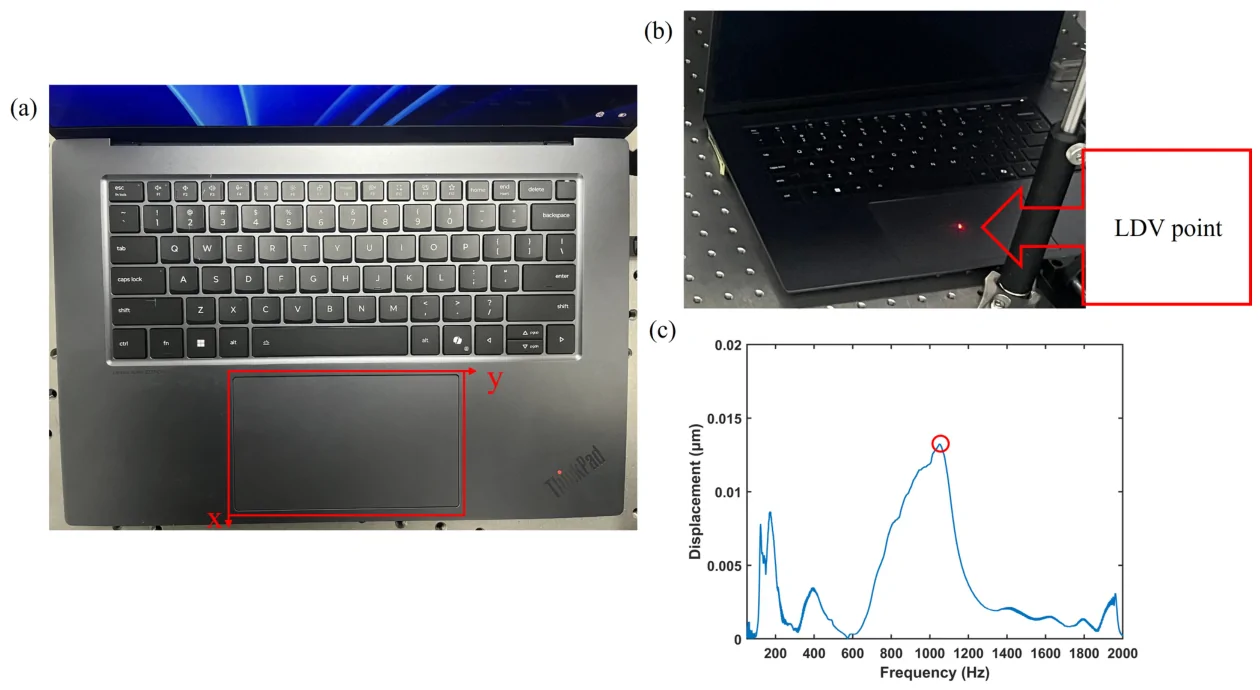
Experiment on a Lenovo ThinkPad laptop touchpad: (**a**) measurement region (red box) and image-coordinate directions; (**b**) LDV measurement point; (**c**) Frequency-response spectrum of the ThinkPad touchpad measured using the LDV. The red circle marks the approximately 1051 Hz peak selected for full-field single-frequency vibration measurement.

To verify the applicability of the developed product in a practical scenario, the EMODE-1 measurement system was used to test the touchpad of a Lenovo ThinkPad laptop (Lenovo, Shanghai, China). The dynamic response of a touchpad can affect the haptic feedback, vibroacoustic quality, and structural reliability of the device. Therefore, the region with the largest vibration response (outlined by the red box in Figure 13a) was measured, and its corresponding amplitude was obtained. Such analysis can help reduce discomfort or undesirable haptic feedback caused by vibration during user interaction. A linear sweep signal from 0 to 2000 Hz was first generated in MATLAB R2025a and applied acoustically through the built-in laptop loudspeaker. The LDV recorded the response at the location shown in Figure 13b. Figure 13c shows a pronounced response peak at approximately 1051 Hz; this frequency was therefore selected for full-field single-frequency vibration measurement.

**Figure 14:**
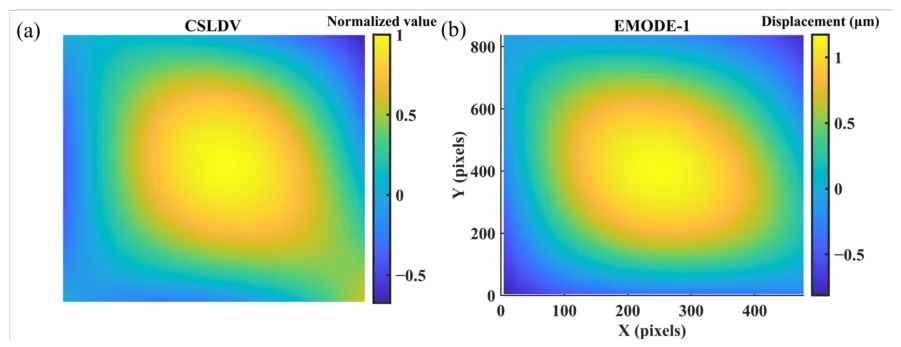
Comparison of the measured mode shapes at 1051 Hz. (**a**) CSLDV measurement result, normalized to show the mode-shape pattern; (**b**) EMODE-1 measurement result, with displacement shown in μm.

Under 1051 Hz acoustic excitation, the touchpad mode shape was measured using EMODE-1 and CSLDV, with the CSLDV result used as the comparative reference. Figure 14 shows that the dominant mode-shape distributions obtained by the two methods are consistent, with a MAC value of 0.8878. The differences are concentrated mainly in the weak-vibration regions near the edges, possibly because of poor local speckle quality and low signal-to-noise ratio. This experiment demonstrates that the proposed method can not only recover small-amplitude, high-frequency vibrations on regular specimens but also measure operational deflection shapes on practical, complex surfaces. Repeated measurements under the same operating and excitation conditions yielded closely consistent results, supporting short-term response repeatability under the tested conditions. However, operating temperature was not monitored and its influence on the response was not evaluated; the method therefore still requires sufficient response stability throughout acquisition, and its robustness to temperature variations remains unverified.

Beyond the experiments presented here, the EMODE-1 system has potential applications in steady-state vibration testing of UAV blades and vehicle structures. Under controlled periodic excitation, it could characterize full-field blade vibration or the vibration-induced deformation of vehicle structural components, supporting dynamic model validation and vibration-reduction design. Such applications require optical accessibility, suitable image texture, and sufficient response repeatability throughout acquisition.

## 5. Conclusions

This study presented a full-field vibration measurement method integrating stroboscopic equivalent-time sampling with physics-guided spatiotemporal noise decoupling. For a plastic plate vibrating at 251.1 Hz, a low-frame-rate acquisition of approximately 8.34 fps yielded a reconstructed mode shape with a MAC value of 0.9233, comparable to 0.9225 obtained using a high-speed camera. The pulse-width experiment at 7154 Hz demonstrated the importance of sufficiently short illumination pulses for limiting motion blur. Integration into the EMODE-1 system further enabled touchpad vibration measurement at 1051 Hz, achieving a MAC value of 0.8878 relative to CSLDV.

The method enables high-frequency periodic vibration measurement using low-frame-rate stereo cameras while preserving spatial resolution. Its applicability depends on sufficient response repeatability and controlled sampling timing throughout acquisition; it is not intended for the direct reconstruction of arbitrary transient or random vibrations.

Future work will explore acquisition and reconstruction strategies for non-stationary vibrations to address the current reliance on cycle-to-cycle repeatability. The feasibility of replacing the current spatial filtering with polynomial fitting will also be investigated, with particular attention to noise suppression, amplitude fidelity, and the preservation of local mode-shape details.

## Figures and Tables

**Table 1:** Parameters of the high-speed and low-speed cameras.

Model	Resolution (Pixels)	Frame Rate (fps)	Price (CNY 1000)
MV-CH050-10UM	1168 × 1200	8.342193	6
CP70-1HS-M/C-1900	832 × 840	2511	160

**Table 2:** Specifications of the EMODE-1 Measurement System.

Specifications	Standard Configuration	Custom Configuration
Image pixel resolution	Up to 2000 × 2000 pixels	Customizable
Image measurement resolution	Up to 80 μm	—
Measurement frequency range	1–10 kHz	Customizable
Working distance	500 mm	Customizable
Field of view	160 mm × 160 mm	Customizable
Output physical quantities	Displacement, velocity, and acceleration, selectable via software	—
Frequency measurement range	1–10 kHz	Customizable
Measurement resolution	sub-10 nm displacement sensitivity under optimized conditions	—
Signal source mode	Outputs a 1–10 kHz sinusoidal signal	Customizable
External-input-guided mode	Supports external vibration-signal input from 1 to 10 kHz; compatible with single-point laser Doppler vibrometers, accelerometers, etc.	Customizable
Control mode	Software control	—
Output data format	CSV; the software can directly display the mode shape within a selected region	—
Software	Windows-based software for displaying, storing, and analyzing measured modal data	—
Overall dimensions	365 mm (L) × 170 mm (W) × 120 mm (H), excluding the high-speed stroboscopic light source	—

## Data Availability

Data will be made available on request.
